# Periodic Arrays of Plasmonic Ag-Coated Multiscale 3D-Structures with SERS Activity: Fabrication, Modelling and Characterisation

**DOI:** 10.3390/mi15091129

**Published:** 2024-09-04

**Authors:** Marta Lafuente, Lucas J. Kooijman, Sergio G. Rodrigo, Erwin Berenschot, Reyes Mallada, María P. Pina, Niels R. Tas, Roald M. Tiggelaar

**Affiliations:** 1 Departamento de Ingeniería Química y Tecnologías del Medio Ambiente, Campus Rio Ebro, C/Maria de Luna s/n, Universidad de Zaragoza, 50018 Zaragoza, Spain; rmallada@unizar.es (R.M.); mapina@unizar.es (M.P.P.); 2 Instituto de Nanociencia y Materiales de Aragón (INMA), CSIC-Universidad de Zaragoza, 50009 Zaragoza, Spain; sergut@unizar.es; 3 Mesoscale Chemical Systems, MESA+ Institute, University of Twente, P.O. Box 217, 7500 AE Enschede, The Netherlands; l.j.kooijman@utwente.nl (L.J.K.); j.w.berenschot@utwente.nl (E.B.); n.r.tas@utwente.nl (N.R.T.); 4 Departamento de Física Aplicada, Facultad de Ciencias, Universidad de Zaragoza, 50009 Zaragoza, Spain; 5 Networking Research Center on Bioengineering, Biomaterials and Nanomedicine, CIBER-BBN, 28029 Madrid, Spain; 6 NanoLab Cleanroom, MESA+ Institute, University of Twente, P.O. Box 217, 7500 AE Enschede, The Netherlands

**Keywords:** silver-coated 3D-structures, pyramids and octahedrons, silicon micromachining, periodic arrays, multiscale dimensions, FDTD simulations, SERS activity

## Abstract

Surface enhanced Raman spectroscopy (SERS) is gaining importance as sensing tool. However, wide application of the SERS technique suffers mainly from limitations in terms of uniformity of the plasmonics structures and sensitivity for low concentrations of target analytes. In this work, we present SERS specimens based on periodic arrays of 3D-structures coated with silver, fabricated by silicon top-down micro and nanofabrication (10 mm × 10 mm footprint). Each 3D-structure is essentially an octahedron on top of a pyramid. The width of the top part—the octahedron—was varied from 0.7 µm to 5 µm. The smallest structures reached an analytical enhancement factor (AEF) of 3.9 × 10^7^ with a relative standard deviation (RSD) below 20%. According to finite-difference time-domain (FDTD) simulations, the origin of this signal amplification lies in the strong localization of electromagnetic fields at the edges and surfaces of the octahedrons. Finally, the sensitivity of these SERS specimens was evaluated under close-to-reality conditions using a portable Raman spectrophotometer and monitoring of the three vibrational bands of 4-nitrobenzenethiol (4-NBT). Thus, this contribution deals with fabrication, characterization and simulation of multiscale 3D-structures with SERS activity.

## 1. Introduction

Research in surface-enhanced Raman scattering (SERS) is a rapidly growing field due to its impressive sensing characteristics in terms of sensitivity and selectivity [1,2]. Research is mainly focused on fundamental theoretical studies to predict and understand the SERS effect, as well as on (practical) development of novel SERS substrates with enhanced properties.

The SERS effect depends mainly on the electromagnetic contribution due to oscillation of electrons in a metallic structure when it is exposed to an external electromagnetic field. The order of magnitude of the electromagnetic SERS enhancement can be in the range of 10^4^ to 10^10^ depending on the metallic structure geometry and its periodicity, thus, the upper end corresponding to specimens with narrow nanogaps or sharp tips [3]. The second contribution to the SERS effect is the chemical enhancement as a result of direct adsorption or proximity of molecules to the metallic surface. This is a molecule-dependent factor which affects the enhancement between 10^−1^ to 10^2^ [4,5].

Theoretical modelling of the electromagnetic properties allows rational evaluation of the SERS activity of designed SERS specimens. The SERS specimen plays a crucial role in the SERS effect, because the electromagnetic enhancement depends on the material(s), size and shape of the structures on the specimen. SERS substrates can be classified in four basic categories [1]: (i) nanoparticles in a colloidal suspension [6]; (ii) bottom-up substrates based on nanoparticles assembled on solid substrates [7], and 3D-micro/nanostructures fabricated on a solid substrate by (iii) top-down [8] or (iv) bottom-up approaches [9]. These categories present significant differences in terms of preparation, homogeneity and uniformity of plasmonic structures, instrumentation and scaling up, as well as the know-how/equipment required for fabrication.

The assembly of the colloidal nanoparticles onto solid substrates entails improved reliability and reproducibility of SERS signals compared to nanoparticles in suspension [7,10]. Different strategies have been used for the deposition of the colloidal nanoparticles onto solid supports. The classical bottom-up methods are chemical assembly through bifunctional molecules [11,12], electrostatic interactions [13,14] assembly by capillarity [15], spin coating [16], or more sophisticated and novel approaches like Langmuir-Blodgett [17] and growing in situ via photodeposition [18]. Nevertheless, none of these approaches reaches the level of uniformity and reproducibility of top-down SERS specimens.

Top-down methodologies offer many options for fabrication of uniform and periodic SERS specimens with a wide selection of sizes and geometries. These methodologies involve lithographic techniques like electron and focus ion beam lithography (EBL and FIB, respectively) [19,20], UV-photolithography [21], nanoimprint lithography (NIL) [22] or displacement Talbot lithography (DTL) [23,24] and other chemical processes. Although these techniques can be applied for fabrication of a variety of geometries, the most studied/realised geometries are vertically aligned nanostructures, i.e., nanopillars and derived geometries [25]. Yue et al. [20] reported gold-coated silicon dimer-nanopillar arrays prepared by EBL, with gaps of 8–10 nm yielding a high enhancement factor (EF), viz. 10^9^ and low standard deviation (3.2–5.6%). The group of Boisen et al. reported alumina/silicon nanohoodoos prepared by block copolymer lithography with gold nanoparticles on the top. These architectures reached an EF greater than 10^7^ with a coefficient of variation of 6% [26]. In addition, this group also fabricated gold coated silicon nanopillars (by maskless dry etching) with an EF in the order of 10^6^ due to the leaning of the pillars upon contact with a liquid [27]. Maskless plasma etching was used by Sim et al. for preparing polyimide nanopillars that were covered by silver nanoparticles (NPs), resulting in an EF of 10^8^ and standard deviation of 7.6% [28]. Recently, our group reported on the use of gold NPs on silicon nanocones (prepared by DTL and reactive ion etching (RIE)) with an EF of 10^7^ and a variance as low as 4% [23]. Liu et al. [29] prepared SU8 nanopillars with gold caps, creating an optical cavity between the top gold caps and the bottom gold layer, which reached an EF of 10^6^ and signal deviation of 6.7%. Nature is also replicated though pillars of diatoms, having an EF of 10^7^ and a modest deviation of <20% [22].

Besides these top-down structures, also top-down fabricated plasmonic pyramids have been reported, but to a lesser extent than vertically aligned nanostructures. Most studies used non-uniform and randomly distributed pyramids prepared by wet etching [30,31,32]. For example, recently De Sousa Junior et al. [33] reported non-uniform arrays of micro-pyramids (height of 2–4 µm; prepared by maskless wet etching) that, upon coating with AgNPs, reached an EF of 10^8^. Another approach is use of a silicon substrate with inverted pyramids as mold for preparing polymeric SERS specimens based on pyramids [34,35]. In this way, Wang et al. [36] created ordered polymeric pyramids (width ~3 µm) coated with AgNPs, giving an EF of 10^7^ and deviation of 5.8%. Likewise, Zhang et al. fabricated two- and three-dimensional micro/nanopyramids arrays on PDMS substrates by combining the tip-based force modulation indentation method with the reverse nanoimprinting process [37]. After gold-coating, these pyramids (height ~230 nm and spacing ~1 µm) reached an EF of 6 × 10^6^. Das et al. compared inverted and upright nanoyramids (width ~1 µm; depth and height ~650 nm and ~500 nm, respectively) on PDMS, coated with Ag/Au: inverted nanopyramids yielded a higher amplification than upright structures, viz. 3.9 × 10^6^ vs. 7.9 × 10^5^, respectively [38]. Jin et al. reported an EF of 10^6^ for gold nanopyramids (width ~200 nm and spacing ~2 nm), due to the strong charge coupling between adjacent nanopyramids [39].

For some of the above described specimens the origin of the SERS amplification is the enhancement originating from the nanoparticles coated on the pyramids. In contrast, in case of specimens based on pyramids covered with a continuous metallic thin film a second contribution to the SERS enhancement is possible (i.e., resonance modes), e.g., silicon nanopyramids (width ~350 nm) covered with thin films of graphene and gold enhanced the Raman signal on the tip of the gold-coated pyramid (EF ~10^9^) [40]. This effect is also acting in case of Ag/Au-coated ordered arrays of fractal microstructures: to the best of our knowledge our previous work was the first article where arrays of 3D-microstructures were used as a SERS active platform [41]. These microstructures were formed by iterative steps consisting of selective anisotropic wet etching of silicon and corner lithography, followed SiO_2_ deposition, anodic bonding to a glass substrate, dissolution of the silicon substrate, deposition of a continuous silver layer and coating with AuNPs; such SERS platforms reached an EF of 10^5^ due to the contribution of the plasmonics modes supported by the own Ag layer and the gaps between the Ag layer and Au nanoparticles.

In this work, we made a step forward by tuning the width of the octahedron (G1) on top of the pyramidal (G0) microstructure from the microscale to nanoscale. Specimens with periodic arrays of Ag-coated 3D-structures with G1-widths of 0.7 µm, 1 µm, 2 µm and 5 µm were fabricated and their SERS activity characterised. The motivation for downscaling G1 to values below 5 µm (as used in [41]) followed from simulations. Moreover, implementation of reduced G1-values makes it possible to use smaller G0-widths, leading to a higher amount of 3D-structures on a given footprint which on its turn benefits the SERS activity of the specimens. The fabrication procedure is based on UV-lithography, selective wet etching of crystalline silicon in combination with ‘oxide-only’ corner lithography and, as final step, deposition (by means of e-beam evaporation) of a thin film of silver on the created 3D SiO_2_-structures. The SERS activity of arrays of multiscale 3D-structures—also referred to as G1/G0-arrays—is assessed through the analytical enhancement factor (AEF) using the non-resonant molecule 4-NBT. Moreover, the sensitivity of the various arrays was evaluated using a portable Raman spectrophotometer to mimic conditions close to an in-field test. Finally, the origin of the SERS amplification of Ag-coated periodic G1/G0-arrays is studied by FDTD modelling.

## 2. Materials and Methods

### 2.1. Materials

4-nitrobenzenethiol (4-NBT, 80%) was obtained from Merck (Darmstadt, Germany). Absolute ethanol (99.9%) was purchased at VWR Chemicals (Amsterdam, The Netherlands).

### 2.2. Computational Simulations

A custom finite-difference-time-domain (FDTD) code [42] was utilized to compute electromagnetic (EM) fields in the Ag-coated periodic G1/G0-arrays. When plasmonic isolated scatterers are periodically arranged, such as in the G1/G0-arrays, the EM resonances typically result from a hybridization of localized plasmonic resonances at the scatterers (which acts like meta-atoms) and surface plasmons, which mediate the EM coupling among them. To differentiate the surface-like response from the EM modes localized at the G1/G0-structures objects, isolated systems were also considered in simulations. The computational domain was finished with absorbing boundary conditions to avoid spurious back-reflections. In the periodic structures, a unit-cell of the G1/G0-arrays is necessary for calculations, imposing Bloch’s theorem along the directions defined by the lattice vectors. The dielectric constant of silver, obtained from tabulated data [43], was incorporated into the FDTD model using a Drude-Lorentz approach [44]. Structures were illuminated by plane-wave at normal incidence. A sufficiently fine mesh was employed to accurately represent the EM fields within the metal (see [42] for further details).

The average SERS gain for a given structure S, denoted $\alpha_{S}$, is defined as:

(1) $$\alpha_{S}=\langle {\left|{\vec{E}}_{S}\left(\vec{r},\lambda \right)/{\vec{E}}_{inc}\left(\vec{r},\lambda \right)\right|}^{4}\rangle$$

where the brackets indicate averaging over the SERS-generating volume. In the numerical implementation of Equation (1), the electric field intensity at the metal surface (the source of SERS) is approximated by averaging the fields *Ē_S_* within each mesh cell in contact with the metal. The incident field *Ē_inc_* is also obtained in vacuum at the same position $\vec{r}$ and wavelength *λ*, to ultimately reproduce the local SERS intensity.

Throughout this work, the lattice constant is 4.6 µm (for G0) for periodically arranged G1/G0-structures. Note that the calculations were performed assuming a square lattice, whereas most experimental samples were fabricated with a hexagonal arrangement. This approach reduces the computational burden without affecting the results of the SERS gain. The thicknesses of the metal and silicon dioxide are 46 nm and 78 nm, respectively.

### 2.3. Fabrication of Ordered Arrays of Ag-Coated Multiscale 3D-Structures

**Figure 1:**
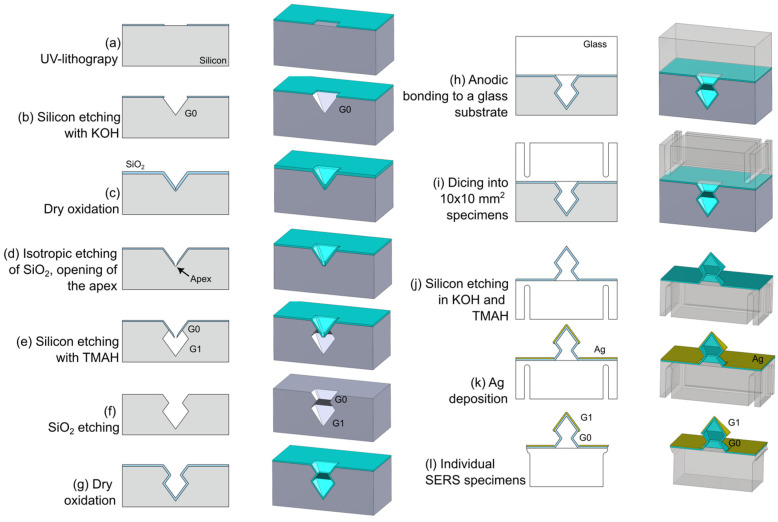
Schematic representation (in 2D and 3D) of the fabrication of SERS active specimens composed of periodic arrays of SiO_2_ multiscale 3D-structures.

In this subsection, a general description of the fabrication of the SERS active specimens based on Ag-coated 3D-structures is given, including a sketch-wise cross-sectional representation at various stages of the fabrication process (Figure 1). Details of the fabrication sequence are provided in Appendix A.

Mould-structures for the 4 different G1/G0-arrays are made in (100)-oriented silicon substrates. Post to dry oxidation, by means of UV-lithography array patterns of circular openings are defined photoresist, which are transferred into the underlying SiO_2_-film (Figure 1a). After removal of the photoresist, selective etching of silicon in potassium hydroxide (KOH) is performed, yielding inverted G0-micropyramids with a base of 2 µm or 5 µm (Figure 1b). Post to cleaning the SiO_2_ hard mask is removed, followed by dry oxidation. Due to retarded oxidation at apices, viz. the concave corner of the inverted pyramids, the SiO_2_-film will be thinner at these apices compared to crystal planes (Figure 1c). By using timed isotropic etching in 1% hydrofluoric acid (HF) to reduce the thickness of the SiO_2_-film, the oxide in the apex can be removed (providing access to the underlying silicon) whereas SiO_2_ remains as a mask on the (111)-Si and (100)-Si planes (Figure 1d). Subsequently, the exposed silicon at the apex is selectively etched in tetra-methyl-ammonium-hydroxide (TMAH), yielding an octahedral feature at this location (Figure 1e). The etch time in TMAH determines the width of this G1-octahedron (see Table A1). Next, the SiO_2_-mask is removed, which completes the G1/G0-structures that serve as moulds for the 3D-structures (Figure 1f). The next step is growth of a SiO_2_-film of 75 nm by dry oxidation (Figure 1g), which is followed by anodic bonding of the silicon substrate to a Mempax glass substrate (Figure 1h). Subsequently dicing into the glass-side of the Si-glass stacks is performed (Figure 1i) on a 10 mm × 10 mm grid, which is the footprint of the SERS active specimens. Then the silicon is dissolved by means of wet-etching in KOH and TMAH (Figure 1j). This is followed by deposition of ~40 nm silver (Ag) on the SiO_2_ 3D-structures by means of evaporation (Figure 1k). As a final step, the grid of diced-in lines is used to manually release individual specimens (Figure 1l). It is worthy to note that the SiO_2_ 3D-structures were cleaned by immersion in Piranha solution (3:1 H_2_SO_4_: H_2_O_2_) for 20 min at 80 °C, rinsed in DI-water and N_2_-dried before Ag deposition. Ag-coated arrays with 4 different G1-dimensions are realised, i.e., octahedrons with widths of approx. 0.7 µm, 1 µm, 2 µm, and 5 µm, named as G1-0.7, G1-1, G1-2 and G1-5, respectively.

### 2.4. Imaging

Scanning electron microscopy (SEM) images were recorded using a FEI INSPECT 50 (20 kV and spot size 3.5). With ImageJ analysis software (v1.0), five to ten SEM images of each specimen were analysed to obtain dimensional information (*N* > 100 measurements). The uncertainty is expressed in ±1σ.

### 2.5. Optical Characterization

Optical characterization of specimens was performed with a Vis-NIR spectrophotometer (PerkinElmer Lambda 950 UV-vis-NIR) over the 500 to 1000 nm range, employing an integrating sphere. The transmittance (*T*) and the reflectance (*R*) spectrum were collected separately, and used to calculate the absorptance spectrum of the SERS substrates based on silver-coated 3D microstructures. The absorptance (*Abs*) spectra were calculated as *Abs* = 100 − (*T* + *R*).

### 2.6. Raman and SERS Measurements

Raman spectra were acquired using two Raman spectrophotometers, i.e., a high-resolution system (WITec) and a portable, handheld system (Serstech) with a limited spectral resolution. Firstly, SERS activity and SERS maps were recorded with a confocal Alpha300 Raman spectrophotometer from WITec (Ulm, Germany) with a spectral resolution of 2 cm^−1^. All measurements were made in backscattering mode with an excitation wavelength of 785 nm. The microscope objective was 20× (1.92 µm of spot diameter) for G1-2 and G1-5 specimens, and 50× (1.13 µm of spot diameter)) for G1-0.7 and G1-1 specimens. The laser power was 1 mW and the acquisition time 1 s. Different maps of 50 µm × 50 µm or 15 µm × 15 µm containing 100 measurement locations were measured on each SERS specimen. 4-nitrobenzenethiol (4-NBT) was selected as probe molecule and the Raman intensity of its NO_2_ symmetric stretching vibrational mode at ~1338 cm^−1^ was used as a mapping signal. The relative standard deviation (RSD) was calculated averaging the spots from the map which reported an amplification of the signal. As reference, planar specimens were used, being a flat borosilicate glass surface (without the 3D-structures) coated with 40 nm of silver. The Raman spectrum of liquid solution (10 mM) of 4-NBT was measured using 60 mW and 50 s. In all SERS and Raman spectra automatic baseline (background) subtraction was done by WITec Software 2.10.

The sensitivity of some of the specimens was assessed through the limit of detection (LOD) with a portable handheld Raman spectrophotometer, i.e., the Serstech 100 Indicator (Serstech, Lund, Sweden), with a spectral resolution of 10 cm^−1^. All measurements were made in backscattering mode with an excitation wavelength of 785 nm, exposing the specimen through an optical fiber (30 µm of spot diameter) and applying a power of 165 mW for 15 s. This spectrometer, which has a wider field of view, captures the spectrum of several Ag-coated 3D-structures in parallel. Ten different spectra were recorded and averaged per specimen, and three specimens were measured and averaged per 4-NBT concentration. Vibrational modes shifted to 1099 cm^−1^, 1338 cm^−1^ and 1575 cm^−1^ and assigned to C-H, NO_2_ symmetric stretching and C-C stretching [45], respectively, were monitored. A Raman spectrum of pure powder of 4-NBT was measured using 165 mW and 10 s. For all spectra recorded by Serstech equipment, automatic baseline (background) subtraction was carried out by the Serstech software (v5.14).

### 2.7. Calculation of Analytical Enhancement Factor (AEF)

The AEF provides quantitative information of the signal enhancement that can be expected from a specific SERS specimen with respect to a reference Raman experiment [3,46]. The 4-NBT NO_2_ mode displayed at 1338 cm^−1^ was used for monitoring. The AEF was calculated using Equation (2):

(2) $$AEF=\frac{\frac{I_{SERS}^{*}}{C_{SERS}}}{\frac{I_{Raman}^{*}}{C_{Raman}}}$$

where *C_Raman_* and *C_SERS_* are the 4-NBT concentrations during the Raman measurements and SERS conditions, respectively. I*_SERS_ is the normalised intensity of 4-NBT molecules on the SERS specimen, and I*_Raman_ corresponds to the normalised intensity of 4-NBT molecules measured in the liquid phase (10 mM). The solution was placed in a polytetrafluoroethylene (PTFE) container of 5 mm × 5 mm × 5 mm (length, width and depth, respectively). For SERS measurements, the specimens were incubated in 1 µM of 4-NBT, prepared from the stock solution, in vertical position for 2 h; then, they were rinsed in ethanol and air-dried. Thus, *I_SERS_* is the intensity of the 4-NBT molecules adsorbed on the SERS active specimen. In this work, the SERS spectra of the specimens were measured in five different random areas (50 µm × 50 µm, 100 excitations points/area) and the intensity of the peak at 1338 cm^−1^ was averaged. In order to perform a fair comparison, both intensities were normalised to laser dose and time (cts cm^2^ mW^−1^ s^−1^).

### 2.8. Calculation of Limit of Detection (LOD)

The limit of detection (LOD) of Ag-coated arrays was calculated with Equation (3) [47]:

(3) $$LOD=I_{blank}+3\;\sigma_{blank}$$

where *I_blank_* is the averaged SERS intensity of the specimen after immersion in pure ethanol (solvent used for the 4-NBT solutions) in the spectral windows shown in Table A2 and Table A3 (Appendix B) of the blank experiments and *σ_blank_* is the standard deviation of this averaged intensity.

#### 2.8.1. Blank Measurements

For characterization of the noise, three different specimens with Ag-coated G1/G0-arrays were measured before and after incubation in pure ethanol.

#### 2.8.2. 4-NBT Solutions

A stock solution of 4-NBT (10 mM) was prepared in pure ethanol. Then, 4-NBT solutions with concentration of 1000 µM, 100 µM, 10 µM, 1 µM and 0.1 µM were prepared by step-by-step dilution with the same solvent. For SERS measurements, three different specimens of each type of G1/G0-array were incubated in 10 mL of the indicated concentration of 4-NBT in vertical position for 2 h. After that, the specimens were rinsed in pure ethanol, air-dried and SERS characterised.

## 3. Results and Discussion

### 3.1. Modelling of Ordered Arrays of Ag-Coated Multiscale 3D-Structures

**Figure 2:**
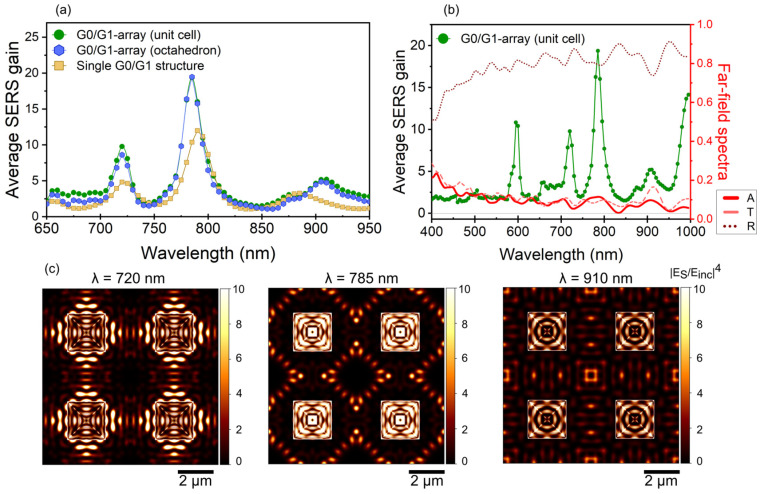
(**a**) Average SERS gain calculated using Equation (1) for the G1 = G0 ~2 µm structure. The green and blue lines show the calculations for an array with a lattice constant of 4.6 μm, where the SERS signal is averaged over the entire unit-cell and the octahedron, respectively. For comparison, the calculations for an isolated structure are depicted with orange symbols. (**b**) SERS gain and transmission, reflection and absorption ranging from visible to near infrared wavelengths. (**c**) Top-view maps of local SERS gain at the EM resonances appearing in (**a**). Four unit-cells are depicted. All the averaged values for the lattice shown in (**a**) were calculated from these and additional maps at different wavelengths. Both the corresponding wavelength and averaged SERS is indicated as subtitles above each figure. The thicknesses of Ag and SiO_2_ are 46 nm and 78 nm, respectively.

Figure 2a shows the SERS gain calculated using Equation (1) for the G1 = G0 ~2 µm structure. The green and blue lines show the calculations for an array with a lattice constant of 4.6 μm, where the SERS signal is averaged over the entire unit-cell and the octahedron, respectively. For comparison, the result for an isolated structure is depicted with orange symbols (integration over the octahedron). The three most intense resonances correspond to the excitation of plasmonic modes strongly confined on the octahedrons, as they appear in both configurations at similar spectral locations, anticipating that the SERS signal mainly originates from localized resonances. This is confirmed when comparing the SERS gain obtained from the entire surface to the signal generated at the octahedron.

Clearly, the contribution to SERS gain from the surface is negligible. Our theoretical analysis of SERS gain and absorption also reveals that the plasmonic resonances extend from the near-infrared (IR) to the visible (VIS) spectrum (Figure 2b). Most SERS devices do not allow for the spectroscopic interrogation of the near-field. Therefore, it is challenging to assess the existence of plasmonic resonances through SERS alone. In contrast, techniques like energy loss spectroscopy (EELS) can map the EM resonances with high precision, but this comes at a higher cost (and the multi-scale 3D-structures cannot be easily measured in a Transmission Electron Microsope). As shown in Figure 2b, absorption derived from far-field calculations of transmission and reflection provides evidence of near-field resonances that are accessible for the SERS process.

Top-view maps of the local SERS gain are shown in Figure 2c, depicting ${\left|{\vec{E}}_{S}/{\vec{E}}_{inc}\right|}^{4}$ on a four unit cell area at the three most intense EM resonances. Numerical integration of these maps lead to the SERS values shown in Figure 2a. The average SERS gains are 9.7, 19.9 and 5.2 at respectively 720 nm, 785 nm and 910 nm.

We attribute the EM modes accessible in the systems to Surface Plasmon Polaritons (SPPs) and Localized Surface Plasmons (LSPs). SPPs are collective oscillations of free electrons on a metal surface coupled to incident light (plasmons). Coupling of light with SPPs requires conservation of frequency and momentum. However, since incident propagating light has a small momentum that of SPPs, they can only be excited by introducing ‘defects’ on the metal surface [48]. A metal surface drilled by holes or decorated with particles can sustain SPPs. If the holes or particles are periodically arranged, excitation of SPPs can be only along very specific directions, determined by the reciprocal lattice vectors allowed by the system’s band structure. LSPs are similar, but in this case, plasmons are confined to a small region of space, such as a metallic nanoparticle, when it interacts with light. Both kind of modes generate strong EM fields near the metal, leading to enhanced optical phenomena like increased scattering or absorption of light [49]. In our case, the wavelength range is much smaller than the array period, so high-order reciprocal lattice vectors are available that explain the intricate standing wave-pattern on the flat areas (viz. in-between G1/G0-structures) visible in Figure 2c. The LSPs in the Ag-coated periodic G1/G0-arrays exhibit near-field signatures characteristic of either edge or breathing modes, similar to those reported by Campos et al. [50]. Edges behave like very narrow wedges, with the EM modes displaying a similar optical response characterized by extraordinary field enhancement and deep confinement to the metal [51]. Breathing modes can be better understood as cavity modes of the G1-octahedron, as confirmed by the observed standing waves formed on the surfaces of the octahedrons (Figure 2c).

**Figure 3:**
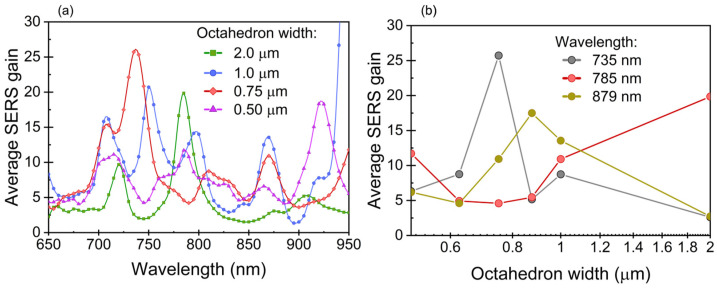
(**a**) Average SERS gain as a function of wavelength for G0 = 2 μm and different octahedron widths (G1). (**b**) the SERS gain at three selected wavelengths as a function of the octahedron width.

Thus, Ag-coated periodic G1/G0-arrays exhibit controllable EM “hot” regions, which are relevant for SERS. More importantly, varying the octahedron size allows for precise tuning of the SERS signal, offering exceptional control over its intensity and characteristics. A numerical study on the dependence of the SERS gain on the octahedron width is shown in Figure 3. The range of G1-widths investigated underscores the versatility of these structures as plasmonic platforms, particularly for sensing applications. The inset shows the SERS gain at three selected wavelengths as a function of the octahedron width. This information is relevant upon discussing the experimental results on SERS.

### 3.2. Fabrication of Ordered Arrays of Ag-Coated Multiscale 3D-Structures

**Figure 4:**
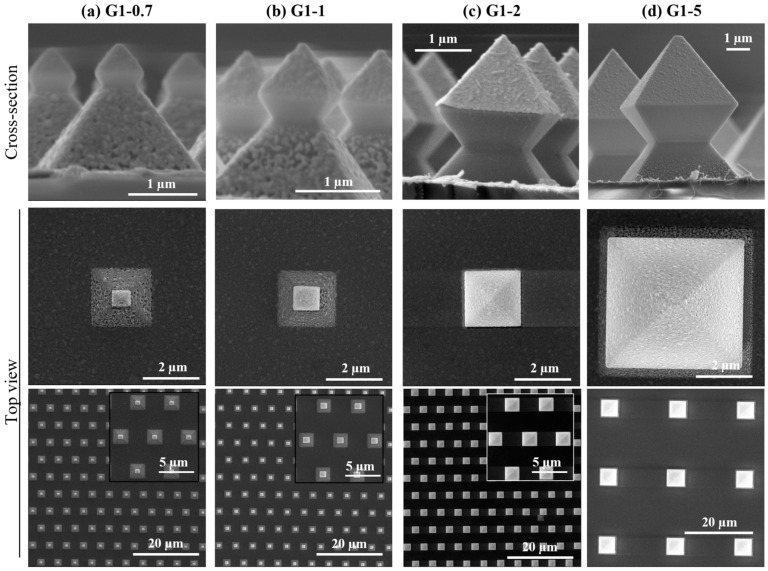
Side-view and top-view SEM-images of SERS active specimens investigated in this work, which have the following widths of the octahedron (G1) and pyramid (G0): (**a**) G1 ~0.7 µm, G0 ~2 µm; (**b**) G1 ~1 µm, G0 ~2 µm; (**c**) G1 = G0 ~2 µm; (**d**) G1 = G0 ~5 µm.

The SERS active specimens in this work are periodic arrays of 3D-structures composed of SiO_2_ on a glass substrate that are coated with a thin film of silver, fabricated following the process sequence as described in Section 2.3. Each Ag-coated 3D-structure is in essence an octahedron (G1) on top of a micropyramid (G0) that are made using corner lithography in combination with selective anisotropic etching of silicon, a fabrication process about which our group previously reported [41,52,53,54]. Figure 4 shows SEM images of cross-sections and top views of the obtained Ag-coated multiscale G1/G0-arrays, which evidences the reproducibility of the fabrication process for these 3D-structures. As can be observed, G1-widths of 0.7, 1 and 2 µm are positioned on G0-pyramids with a base of approximately 2 µm (Figure 4a–c) and a pitch of 5 µm in a hexagonal arrangement. A G1-width of 5 µm is located on a G0-pyramid with a base of 5 µm and a pitch of 20 µm (square arrangement, Figure 4d). SERS active G1/G0-arrays with G1 = G0 = 5 µm were investigated previously by us, and exhibited promising SERS activity [41]. In contrast to our previously reported SERS active platforms [41], in this work the so-called ‘oxide-only’ corner lithography route is employed for the fabrication of the various periodic G1/G0-arrays [54], which is easier/faster since it doesn’t require use of LPCVD low-stress silicon-rich silicon nitride (SiRN) as building block (as used in [41]). The realised dimensions of the fabricated G1/G0-arrays are summarised in Table 1.

In fact, the obtained widths for G0 (pyramid) and G1 (octahedron) slightly deviate from targeted values, i.e., deviations between the targeted width and realised widths are 16%, 9%, 3% and 4% for G1-0.7, G1-1, G1-2 and G1-5, respectively. In fact, the octahedrons do not have a square base (i.e., the G1-widths in *x*- and *y*-directions differ slightly, creating a slightly rectangular base as can be seen in Figure 4 and Table 1), neither the pyramids. The reason for these non-square bases of G1 and G0 originate from the mask employed for UV-lithography: the mask didn’t contain perfect circular openings, but the openings were slightly oval/elliptical. These non-circular openings were transferred into the hard mask (SiO_2_), and led to inverted micropyramids with a rectangular base (rather than square) upon anisotropic etching of silicon. As a consequence of this rectangular base, the realised opening at the apex of the inverted micropyramids will be rectangular as well, leading to small differences in *x*/*y*-widths of the octahedron.

Due to directional deposition, the Ag layer is only present on the top of the octahedrons as well as on (parts of) the pyramids: the ‘neck’ below the octahedrons and pyramids is not covered with Ag. The used e-beam evaporation method has highly directional Ag-flux, allowing the octahedrons to act as a shadow mask, thereby avoiding deposition of Ag onto the bottom part of the octahedrons. This break/discontinuity in the Ag layer between the top and bottom part is the origin of plasmonic modes capable of enhancing the incoming electromagnetic field, as shown in Section 2.2.

**Figure 5:**
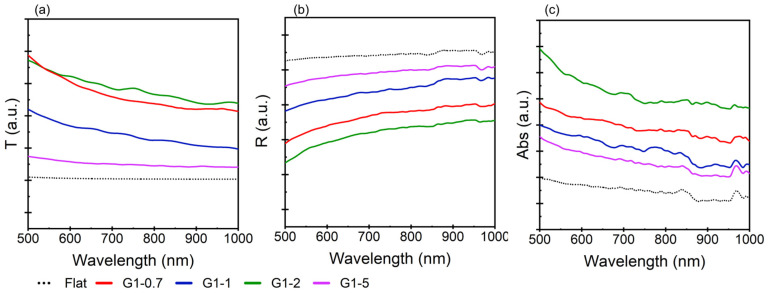
(**a**) Vis-NIR transmission, (**b**) reflection and (**c**) absorption spectra of a flat surface, and specimens G1-0.7, G1-1, G1-2 and G1-5 as experimentally measured.

Figure 5 shows the Vis-NIR absorptance spectra calculated from the measured transmission and reflection spectra. The Ag-coated periodic G1/G0-arrays exhibit a far-field optical response similar to that of a flat metal surface coated with an identical thickness of metal. The key difference is the presence of “ripples”, which indicate the excitation of plasmonic modes. However, the far-field response is primarily influenced by the extensive flat regions between the structures. This is particularly evident for the G1-5 structures. This flat-metal-like behavior is also observed in the numerical calculations, as shown in Figure 2b.

### 3.3. SERS Activity of Ag-Coated Multiscale 3D-Structures

**Figure 6:**
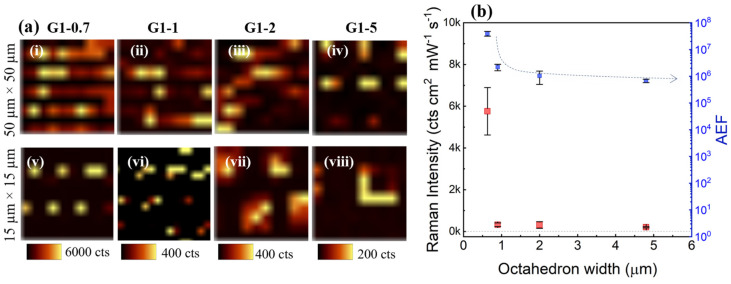
(**a**) SERS mapping of 50 µm × 50 µm (i–iv) and 15 µm × 15 µm (v–viii) obtained by monitoring the peak of 4-NBT at 1338 cm^−1^ for SERS specimens G1-0.7; G1-1; G1-2 and G1-5. (**b**) Effect of width of octahedron on SERS intensity (red) and SERS enhancement (blue) results of Ag-coated G1/G0-arrays. The error bars indicate standard deviation.

The SERS signal of 4-NBT was studied in order to evaluate the SERS activity of the Ag-coated multiscale 3D-structures. 4-NBT is a non-resonant molecule and well-known SERS target analyte for the quantification and comparison of enhancement factors [55]. We first investigated the capability of the plasmonic SERS specimens for improving the Raman signal of 4-NBT by recording SERS intensity maps on the various Ag-coated G1/G0-arrays. Figure 6 shows representations of these SERS intensity maps monitoring the NO_2_ peak of 4-NBT shifted at 1338 cm^−1^, where the bright areas correspond to the 3D-structures enhancing the Raman intensity.

These mappings reveal a very good spatial resolution matching with the spatial distribution of the Ag-coated 3D-structures on the specimen. As can be observed, the G1/G0-arrays induce the SERS amplification of the signal, demonstrating the high uniformity/reproducibility of the specimens and the fabrication process. It is worthy to note that signal amplification only occurs when the laser spot perfectly matches with a G1/G0-structure. Therefore, for each specimen the *z*-position of the objective was optimised for reaching the maximum amplification of the signal. Nevertheless, this amplification also depends on the *x*-*y* movement of the stage, for this reason, in some rows a few G1/G0-structures are missing, despite the periodic presence of the 3D-structures on the specimens. Furthermore, when the map size was reduced from 50 µm × 50 µm to 15 µm × 15 µm, while maintaining the same number of measurements (100 spots), the visual amplification at the perimeter of an octahedron increased, Figure 6(ai–avi) and Figure 6(av–aviii), respectively. From these smaller SERS intensity maps, it can be seen that the amplification of the signal occurs at the edges (perimeter) of the octahedron. This phenomenon best visible for larger octahedrons, i.e., G1-2 and G1-5 (Figure 6(avii,aviii)), respectively, where the laser spot is able to record a greater number of points. The origin of this amplification of the signal is explained in Section 3.1.

**Figure A1:**
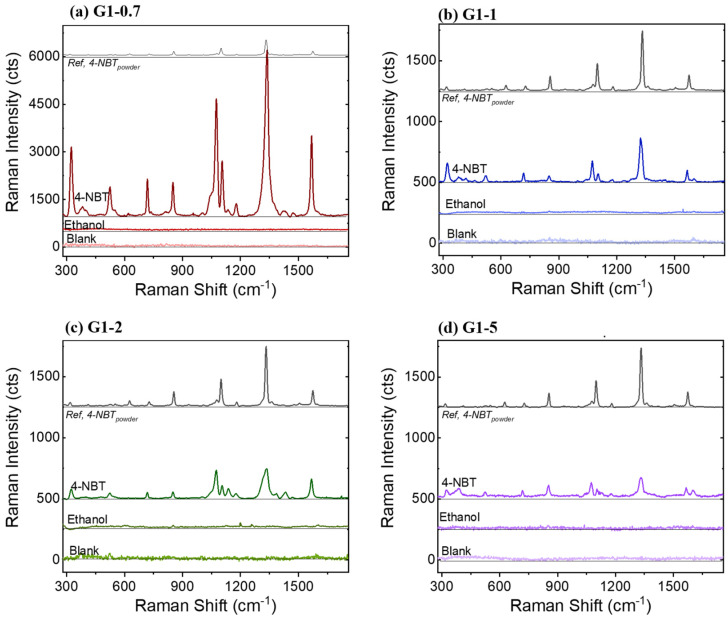
The average SERS spectra of 4-NBT 1 μM obtained on the SERS mappings of (**a**) G1-0.7, (**b**) G1-1, (**c**) G1-2 and (**d**) G1-5; as well as with the Raman spectrum of pure 4-NBT in solid phase and the SERS spectra of each specimen as-fabricated and after immersing in ethanol. Each spectrum is the average of 100 spectra. For better interpretation of the results SERS spectra are displayed with an offset in the *y*-direction.

Figure 6b shows SERS intensity of NO_2_ band of 4-NBT for the various Ag-coated G1/G0-arrays as a function of their octahedron width (G1). Each point represents the averaged intensity of the bright points of the five different maps measured per specimen. As shown in Figure 6b, the SERS signal exhibits a dependence on the width of the octahedron: reduction of the width results in an increase of the SERS intensity. The arrays with the smallest octahedrons (G1-0.7) show a response with a significantly higher SERS intensity than arrays with wider octahedrons. It has to be noted that the RSD of the SERS intensities were 20%, 27%, 40% and 15% for Ag-coated specimens G1-0.7, G1-1, G1-2 and G1-5, respectively. The SERS spectra recorded over the different specimens, as well as the blank experiments of 4-NBT in solid phase, specimens before immersion in 4-NBT solution and after immersion in ethanol (solvent of 4-NBT solution) are shown in Figure A1 (Appendix C). These blank spectra were measured to verify the cleaning process before Ag deposition of the SERS specimens, the purity of the solvent and that the amplification of the signal corresponds to the 4-NBT bands.

The AEF was calculated using Equation (2) and averaging of the bright spots of the mapping results. The results are shown in Figure 6b. Specimens containing the smallest octahedrons show a better SERS response than specimens with wider octahedrons. G1-0.7 achieved an AEF of 3.9 × 10^7^, which is slightly higher than the in general reported AEFs using non-resonant molecules [3,46,55]. This amplification is attributed to the matching of a plasmonic resonance with the excitation wavelength, as indicated by the simulations. Figure 3b shows the SERS gain at three selected wavelengths as a function of the octahedron width. As observed, the trend is not straightforward; SERS does not simply increase with the reduction of the octahedron size: different scenarios arise at different excitation wavelengths. The experimental results can be qualitatively compared to the 735 nm wavelength, despite using a 785 nm wavelength in the experimental setup. Note that spectral shifts are expected when comparing experiments and simulations due to differences in dielectric constants and other sources of discrepancies, such as slight variations in geometry/dimensions. In addition, the uniformity of the near-field around the microstructures may be affected by slight variations in the metallization of G1/G0-structures. Breathing modes (standing plasmons excited on the surface of the octahedron) are likely less susceptible to these changes than edge modes. Consequently, this introduces some statistical variability in the optical response, often manifesting as spectral broadening of the resonances and a reduction in peak intensity compared to ideal systems. It is worthy to note that even at 100% of analyte adsorption efficiency, the AEF tends to be lower than the theoretically predicted EF [3], because of the substrate and molecule orientation. In our previous work [41], the AEF for G1-5 was found to be 2.7 × 10^8^, however, for that work measurements were performed using the resonant Raman probe Rhodamine 6G and the specimens were coated with an Ag film that was covered with AuNPs. Therefore, the AEFs reported in [41] and this work are not comparable, because use of resonant molecules can increase the order of magnitude of the AEF up to 10^2^ [4]. Moreover, in our previous work the origin of the signal amplification was the result of a synergistic interaction between the Ag layer and gold nanoparticles, whereas in the current work only an Ag film is applied.

The AEF is easily determined and is more relevant to experiments with analyte initially in solution or gas phase, nevertheless, AEFs are typically in the order of ~10 to 100 times less than EF_max_ as calculated using only the highest SERS intensities recorded from SERS specimens [3,55]. For a complete characterization of the Ag-coated G1/G0-arrays, the EF_max_ was calculated using the 10 highest intensities recorded for each array. The as such determined values for EF_max_ were about one order of magnitude higher than the AEF-values of G1-0.7, G1-1, G1-2 and G1-5, viz. 1.1 × 10^8^, 1.8 × 10^6^, 1.5 × 10^6^ and 1.3 × 10^6^, respectively. In fact, the RSD-numbers also increase to 28% (G1-0.7), 32% (G1-1), 56% (G1-2) and 36% (G1-5), respectively. The explanation lies on the molecules and hot spot distribution [5]. The hot spot is defined as the location where the molecules experience the maximum amplification of the electromagnetic field [24]. Thus, the field enhancement may differ widely depending on the hot spot, because by definition the EF has a fourth-power dependence with the incoming electromagnetic field [3]. Therefore, molecules attached at different locations on the metallic Ag surface can present a distribution of EF. This higher RSD motivates the benefits of averaging larger number of spectra when the SERS specimens are characterised.

Finally, to corroborate that the G1/G0-arrays produce the signal amplification, a flat reference specimen coated with 40 nm of Ag was measured under identical experimental conditions. Only three of five recorded maps showed amplification of the signal. The AEF was calculated using Equation (2) and found to be 4.5 × 10^3^. This value is up to 4 orders of magnitude lower than the lowest value found for an Ag-coated G1/G0-array (i.e., G1-5). This difference confirms the contribution of the 3D-structures to the AEF, especially the discontinuity between the Ag on the top (on the octahedron) and bottom (on the micropyramids), which allows the excitation of edge LSPs, as described in Section 3.1. Taking into account these results, Ag-coated specimens with the smallest octahedrons, i.e., G1-0.7, were selected for additional experiments.

### 3.4. Sensitivity of Ag-Coated Multiscale 3D-Structures

**Figure 7:**
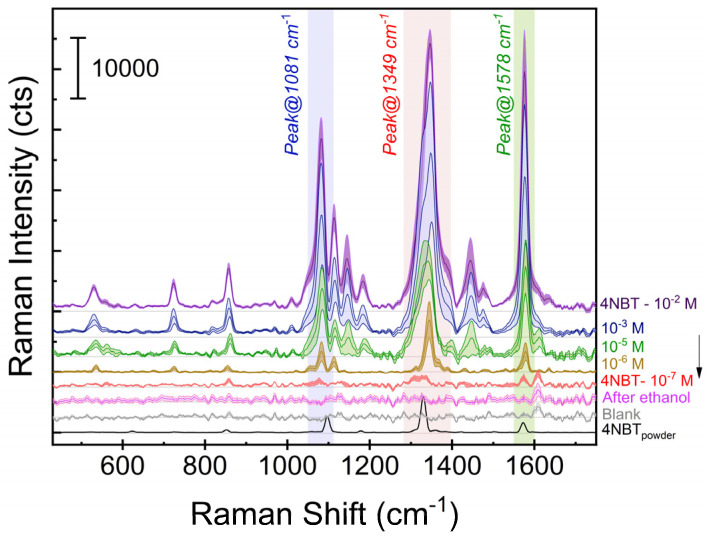
Averaged SERS spectra recorded onto different G1-0.7 specimens (1 specimen per solution) as a function of 4-NBT concentration, together with background and blank SERS spectra (after immersion in pure ethanol) as well as the Raman spectrum of pure 4-NBT (powder). Main lines represent the averaged spectrum and its shadow represents the standard deviation of 10 spectra recorded with the portable Raman spectrophotometer. The vertical coloured-bands indicate the main vibrational modes. For a better visualization, the spectra have been plotted with an offset in *y*-axis.

The LOD is defined as the lowest concentration that a sensor can detect; in this case the sensor is the array of Ag-coated multiscale 3D-structures in combination with the Raman equipment. It is, unfortunately, not common knowledge that the LOD not only depends on the used SERS specimen and the sample concentration, but also on instrumental factors, such as the collection efficiency or the grating efficiency and the detector sensitivity (depends on the cooling system [55]). As such, for under identical conditions of specimen and sample, high-resolution instruments will give a better signal-to-noise ratio than low(er)-resolution systems. Considering future in-field experiments, the LOD of G1-0.7 and G1-5 (for comparison) has been measured using a low(er)-resolution portable spectrophotometer. Another important issue with the LOD based on SERS measurements is the reproducibility. Therefore, following the recommendations of Bell at al. [55] for solid specimens, ten points per specimen distributed over the active area were measured, and SERS data from 3 specimens (measured under identical conditions) were averaged to obtain statistically relevant results. The averaged spectra from G1-0.7 arrays recorded for different concentrations of 4-NBT are shown in Figure 7.

For the complete concentration range (10^−2^–10^−7^ M), the characteristic vibrational bands of 4-NBT at 1081 cm^−1^ (C-H bending), 1349 cm^−1^ (NO_2_ symmetric stretching) and 1578 cm^−1^ (phenyl ring vibration) are clearly distinguishable. Special care was taken to measure the background/noise level of the specimens before and after immersion in pure ethanol (solvent of 4-NBT). Multiple G1-0.7 specimens were measured as-fabricated and after immersion in pure ethanol.

**Figure A2:**
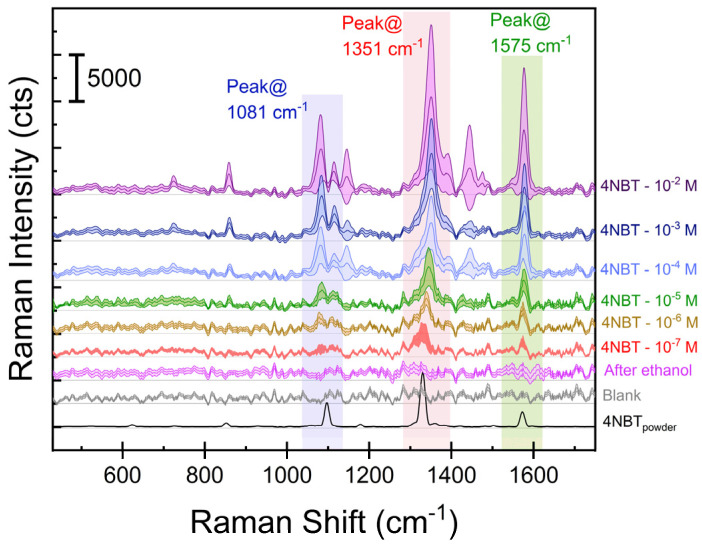
Averaged SERS spectra recorded onto different specimens (1 specimen per solution) with Ag-coated G1-5 arrays as a function of 4-NBT concentration. Background and blank SERS spectra (after immersion in pure ethanol) as well as the Raman spectrum of pure 4-NBT (powder). The main line represents the averaged spectrum and its shadow represents the standard deviation of 10 spectra. The vertical coloured-bands correspond to some characteristic vibrational bands of 4-NBT centred at 1081 cm^−1^ (blue band, attributed to C-H bending), 1351 cm^−1^ (red band, attributed to NO_2_ symmetric stretching) and 1575 cm^−1^ (green shadow, attributed to phenyl ring vibration). For a better visualization, the spectra have been plotted with an offset in *y*-axis.

As can be observed Figure 7, no high intensity background is visible in the spectra recorded on as-fabricated (“blank”) specimens neither after immersion in pure ethanol (“after ethanol”): the recorded SERS signal maintains the same background level along the complete spectral window 400–1750 cm^−1^ (Figure 7). Finally, as expected in terms of absolute value of counts, the spectra recorded using the portable equipment had a higher background compared to the spectra recorded with the high-resolution equipment (see Figure A2 in Appendix D).

**Figure 8:**
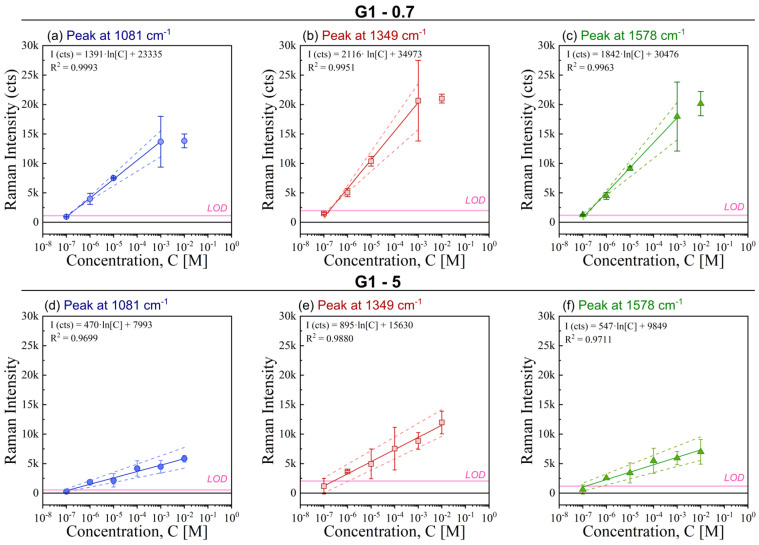
SERS intensity values recorded at 1081 cm^−1^ (blue; (**a**,**d**)), 1349 cm^−1^ (red; (**b**,**e**)) and 1575 cm^−1^ (green; (**c**,**f**)) as a function of 4-NBT concentration for G1-0.7 (**a**–**c**) and G1-5 (**d**–**f**) specimens. Main points represent the average of three different specimens measured under identical conditions and the error bars indicate the standard. The value of the LOD for each peak is included as a horizontal pink line.

A detailed analysis of the SERS response as a function of the 4-NBT concentration is presented in Figure 8. This figure shows the linear fitting of the SERS intensity as a function of the 4-NBT concentration for the three main peaks observed in Figure 7 as recorded on three different G1-0.7 arrays. For best interpretation, the colour of each band in Figure 7 is conserved.

As can be observed, in general for G1-0.7 (Figure 8a–c), when the concentration increases the SERS intensity recorded on the specimen increases. The range with a linear relation between the SERS intensity and 4-NBT concentration is fitted to a linear model given as Equation (4):

(4) $${SERS}_{intensity\;}\left(cts\right)=a\times ln\left({Concentration}_{4-\mathrm{NBT}}\left(M\right)\right)+b$$

where SERS*_intensity_* is the intensity recorded over the specimen, *ln* is the natural logarithm of the 4-NBT concentration, *a* is the slope of the curve and *b* the *y*-intercept. The linear fits obtained for the averaged SERS intensity as a function of 4-NBT concentration are shown in Figure 8 and plotted in different colours for the studied vibrational bands.

The three vibrational bands of 4-NBT recorded for Ag-coated G1-0.7 specimens show a linear relation over the concentration range 10^−7^ M to 10^−3^ M. At higher concentrations (>10^−3^ M), the SERS intensity reaches a plateau and increasing the concentration does not affect the recorded SERS intensity. The origin of this plateau may be that above 10^−3^ M the number of molecules occupy all the active hot spots and upon further increase of the 4-NBT concentration additional molecules can only attach to cold sites. It is worth to note that each concentration was applied to fresh and clean specimens. This plateau may also be the reason for the high RSD at the 10^−3^ M concentration of 4-NBT: the specimens are (almost) saturated, therefore, one specimen could have all the hot spots occupied, whereas this is not the case for (an)other specimen(s). To validate this hypothesis, Ag-coated G1-5 specimens, which contain larger octahedrons (but a lower amount of G1/G0-structures) on the specimen’s footprint were measured under identical conditions (Figure 8d–f and Figure A2). For these G1-5 arrays, the SERS intensity exhibits a linear dependence as a function of the concentration over the studied range, viz. 10^−7^ M to 10^−2^ M. This supports the hypothesis of saturation of hot spots in case of G1-0.7 specimens. In accordance with the previous results (Section 3.3 and Figure 6b), G1-5 arrays exhibit a (much) lower amplification of the signal than G1-0.7 arrays.

The sensitivity of the G1-0.7 and G1-5 specimens was calculated by the limit of detection (LOD) using Equation (3). The calculated LOD-values are depicted in each vibrational panel of Figure 8 as a horizontal pink line. The noise levels are reported in Table A2 and Table A3 (Appendix B). Accordingly, the limit of detection for 4-NBT with Ag-coated G1-0.7 arrays and the used portable spectrophotometer is close to 10^−7^ M for the three main characteristic vibrational bands of this probe molecule. Surprisingly, the most common band used in literature for characterising the SERS effect with 4-NBT, i.e., the band shifted at 1349 cm^−1^, shows the poorest LOD. Similarly, the LOD of Ag-coated G1-5 arrays was calculated and found to be 10^−6^ M, one order of magnitude lower than the LOD of Ag-coated G1-0.7 specimens. This confirms that the G1-0.7 specimens have the best SERS properties.

## 4. Conclusions

SERS active specimens based on Ag-coated multiscale 3D-structures—periodic arrays of octahedrons-on-pyramids (G1/G0-arrays)—were successfully fabricated, with octahedron widths from the nanoscale to the microscale, modelled and characterised. Moulds for the periodic Ag-coated arrays of 3D-structures were fabricated by ‘oxide-only’ corner lithography and selective, anisotropic wet etching of crystalline silicon. Post to a final oxidation step, anodic bonding to a glass substrate and dissolution of the silicon substrate, 40 nm Ag was e-beam evaporated on the SiO_2_ 3D-structures. Four widths—0.7, 1, 2 and 5 µm—of the octahedrons were fabricated by controlling the etching time. The complete Raman fingerprint of 4-NBT was recorded for all realised G1/G0-arrays. The highest amplification of the signal was reached by the smallest Ag-coated 3D-structures, i.e., periodic arrays of 0.7 µm wide octahedrons on 2 µm wide pyramids (G1-0.7), having an AEF of 3.9 × 10^7^ with a RSD below 20%. FDTD modelling indicated that the origin of the amplification was due to the strong localization of EM fields at the edges and surfaces the octahedrons. This strong localization enhances the SERS signal significantly. The excitation of these plasmonic modes, highly sensitive to geometric parameters, provides a tunable platform for optimizing SERS applications. The combination of edge and breathing modes contributes to creating controllable “hot” regions, making these structures highly effective for plasmonic sensing. The sensitivity of specimens was evaluated using a portable Raman spectrophotometer and monitoring the three main vibrational bands. The LOD for the best specimen (G1-0.7) was close to 0.1 µmol L^−1^.

## Figures and Tables

**Table 1:** Dimensions and characteristics of SERS-active G1/G0-arrays studied in this work. The uncertainty is expressed in ± 1σ and *N* > 100 measurements.

Specimen I.D.	Widths G1 [µm]	Widths G0 [µm]	Pitch [µm]	Array Packing
G1-0.7	0.68 ± 0.04	2.20 ± 0.04	4.95 ± 0.04	Hexagonal
	0.58 ± 0.01	2.15 ± 0.04		
G1-1	0.95 ± 0.03	2.08 ± 0.04	4.93 ± 0.03	Hexagonal
	0.88 ± 0.03	2.03 ± 0.04		
G1-2	2.10 ± 0.06	2.28 ± 0.07	4.94 ± 0.04	Hexagonal
	2.02 ± 0.01	2.19 ± 0.08		
G1-5	4.85 ± 0.14	5.58 ± 0.13	19.87 ± 0.11	Square
	4.83 ± 0.08	5.52 ± 0.08		

## Data Availability

The original contributions presented in the study are included in the article, further inquiries can be directed to the corresponding author/s.

## Appendix A

A detailed description of the sequence of steps performed to fabricate the various periodic arrays of 3D-structures is provided in this appendix. Fabrication of the various specimens is done by following this sequence, the difference in configuration (i.e., dimensions) between the four G1/G0-arrays originates from variations in employed deposition and/or etch times. Specific etch/deposition settings for each configuration are listed in Table A1.

**Table A1:** Experimental settings as applied for fabrication of the four multiscale 3D-structures.

Specimen I.D.	Widths G1/G0 [µm]	Pattern of Circular Openings (Diameter [µm]); Pitch [µm]; Packing	KOH Etch Time [min]	Wet Oxidation Time [min]	1% HF Etch Time [min]	TMAH Etch Time [min]
G1-0.7	~0.7/2	1.5; 5; hexagonal	9.5	81	10	16
G1-1	~1/2					21
G1-2	~2/2					43
G1-5	~5/5	5; 20; square	7.5	164	20	140

The mold-structures for the above-listed G1/G0-arrays were made in phosphorus-doped (100)-oriented silicon wafers (100 mm diameter, 380 µm thickness, single side polished, resistivity 1–10 Ohmcm; Okmetic, Vantaa, Finland). Immediately following on ozone-steam cleaning and immersion in 1% hydrofluoric acid (HF, to remove any native oxide; Technic Inc., Saint-Denis, France), substrates were loaded in a furnace for dry oxidation (1100 °C, 95 min; Tempress, Vaassen, The Netherlands) yielding an SiO_2_-film of 164.1 ± 0.8 nm as measured with ellipsometry. By means of UV-lithography a pattern of circular openings was defined in positive photoresist (Olin 907-17; Fujifilm, Tokyo, Japan). In case of G0-pyramids with a base of 2 µm these openings had a diameter of 1.5 µm (in a hexagonal arrangement with a pitch of 5 µm), whereas in case of G0-pyramids with a 5 µm base the openings were 5.0 µm in diameter (in a square arrangement with a 20 µm pitch). The array pattern in the photoresist was transferred into the underlying SiO_2_-film using etching with buffered hydrofluoric acid (3 min; Technic Inc., France) after UV/ozone-exposure of the patterned PR-film (to improve its wettability) and applying a protective resist layer on the backside of the substrate (Olin 908-35; FujiFilm, Japan). The photoresist was removed using 2 baths containing 100% HNO_3_ (2 × 5 min; Technic Inc., France), followed by a dip (30 s) in 1% HF to remove any oxide prior to selective etching of silicon in potassium hydroxide (KOH; Merck, Germany). Formation of inverted micro-pyramids with a base of 2 µm or 5 µm was done in 25 wt.% KOH of 75 °C. The etching was done in sequential time-steps, such that the targeted width of the G0-micropyramids was achieved: in case of a target width of 2 µm the total etch time was 9.5 min, whereas for a width of 5 µm the total etch time was 7.5 min. Post to KOH-etching, substrates were rinsed for 20 min in 70 °C RCA-2 (a 1:1:5 volumetric mixture of hydrochloric acid (HCl; Technic Inc., France), hydrogen peroxide (H_2_O_2_; Technic Inc., France) and demineralised (DI) water) to remove ionic contaminants (i.e., alkali residue), followed by rinsing with DI water, removal of the SiO_2_-film with 50% HF (45 s; Technic Inc., France), another rinsing step with DI water and dry spinning. At this stage the inverted structures being a mould for G0-micropyramids are completed. Post to ozone-steam cleaning (and immersion in 1% HF), substrates were again dry oxidised (1100 °C, 30 min or 95 min; Tempress, The Netherlands) resulting in a SiO_2_-film of 81.3 ± 0.3 nm (G0 = 2 µm) or 164.1 ± 0.8 nm (G0 = 5 µm) as measured with ellipsometry on the (100)-Si surface. Due to retarded oxidation at apices, viz. the concave corner of the inverted pyramids, the SiO_2_-film will be thinner at these apices compared to the flat area of the crystal planes. By using timed isotropic etching in 1% HF to reduce the thickness of the SiO_2_-film, the oxide in the apices can be removed (providing access to the underlying silicon) whereas SiO_2_ remains as a mask on the (111-Si and (100)-Si planes. Immersion in 1% HF lasted 10 min for substrates with inverted pyramids with a base of 2 µm (leaving ca. 35 nm SiO_2_ and ca. 40 nm SiO_2_ on (100)-Si and (111)-Si, respectively), and 20 min for 5 µm (leaving ca. 75 nm SiO_2_ on (100)-Si and ca. 85 nm on (111)-Si). Post to rinsing with DI-water and drying, the substrates were etched in 25 wt.% tetramethyl-ammonium-hydroxide (TMAH, 70 °C; Technic Inc., France) in sequential steps, during which the silicon was anisotropically etched at the apex, yielding an octahedral feature at this location. The total etch time in TMAH depended on the targeted G1 width, and was in the range 16–140 min (see Table A1). Top-view SEM-inspection of the dimensions of the apex (at the bottom of the inverted pyramids) in-between consecutive TMAH-steps provided information about the actual G1-width, based upon which the required etch-time (Table A1) was determined. Following on DI-water rinsing, the SiO_2_-mask was removed by immersion of the substrates in 50% HF for 30 s. At this stage, the G1/G0-structures that are the molds for 3D-arrays are finished. Post to ozone-steam cleaning (including 1% HF immersion) a dry SiO_2_-film of 75.3 ± 0.6 nm was grown on the substrates (1050 °C, 50 min). The oxidised silicon substrate was anodically bonded to a Mempax glass substrate (100 mm diameter, 500 µm thickness, double side polished; Schott, Mainz, Germany) in such a fashion that topside of the silicon substrate containing the G1/G0-structures contacted the glass substrate. Subsequently dicing into the glass-side of the Si-glass stacks was performed to a depth of 340–390 µm (ca. 110–160 µm glass remained; DAD 3220; Disco, Munich, Germany) on a 10 mm × 10 mm grid (which are the footprint dimensions of the SERS active specimens), which was followed by immersion in 50% HF (45 s) to remove the SiO_2_-film from the backside of the silicon. Next, the thickness of the silicon substrate was reduced to 30 µm by etching in KOH (25 wt.%, 75 °C, 304 min). Following on RCA-2 cleaning of the stack (to remove alkali residue, i.e., ionic contaminants), the remaining silicon was removed by etching in 25 wt.% TMAH of 90 °C (26.5 min) and 66 °C (45 min). Afterwards the glass substrate was immersed in DI-water (3 h), followed by cascade-based rinsing in DI-water and spin-drying. Silver was deposited on the SiO_2_ 3D-structures by means of e-beam evaporation (BAK600, Balzers, Liechtenstein, Switzerland). The used settings (2.8 × 10^−7^ mBar base-pressure, 1.1 × 10^−6^ mBar deposition pressure, 0.2 nm/min) resulted in a ~68 nm thick Ag-film on the flat surface in-between the 3D-structures, the Ag-thickness on the planes of the octahedrons exposed to the Ag-flux is ca. 40 nm. The grid of diced-in (but not through) lines in the glass served as manual split lines for releasing individual specimens post to Ag-deposition.

## Appendix B

Three different specimens of Ag-coated G1-0.7 arrays and G1-5 arrays were measured before and after immersion in pure ethanol for being used as the “blank” tests. Ten measurements were recorded per specimen using the portable Serstech spectrophotometer. The SERS spectra of these specimens were carefully analysed before (background) and after immersion in ethanol (blank experiments). Table A2 summarises the main characteristics over the spectral window used for determining of LOD for Ag-coated G1-0.7 arrays, and Table A3 for Ag-coated G1-5 arrays.

**Table A2:** Main characteristics of the spectral windows used for background and blank analysis of Ag-coated G1-0.7 arrays.

Monitoring Peak Centered at [cm^−1^]	Coloured-Band in Figure	Lower Limit [cm^−1^]	Upper Limit [cm^−1^]	Spectral Width [cm^−1^]	Number of Points Averaged
1081	Blue	1027	1096	69	36
1351	Red	1292	1416	124	62
1575	Green	1506	1588	82	24

**Table A3:** Main characteristics of the spectral windows used for background and blank analysis of Ag-coated G1-5 arrays.

Monitoring Peak Centered at [cm^−1^]	Coloured-Band in Figure	Lower Limit [cm^−1^]	Upper Limit [cm^−1^]	Spectral Width [cm^−1^]	Number of Points Averaged
1081	Blue	1026	1144	118	60
1351	Red	1268	1416	148	75
1575	Green	1506	1614	108	55

## Appendix C

SERS spectra of 4-NBT recorded on the different Ag-coated G1/G0-arrays using a high-resolution spectrophotometer are shown in Figure A1.

## Appendix D

SERS spectra of different 4-NBT concentrations recorded on Ag-coated G1-5 arrays using a portable spectrophotometer are shown in Figure A2.
